# Verbal and Musical Short-Term Memory: Evidence for Shared Serial Order Processes?

**DOI:** 10.5334/pb.426

**Published:** 2019-05-16

**Authors:** Simon Gorin, Steve Majerus

**Affiliations:** 1 Psychology and Neuroscience of Cognition Research Unit (PsyNCog), University of Liège, BE; 2 Fund for Scientific Research – FNRS, Brussels, BE

**Keywords:** working memory, serial order, music, language, domain-generality

## Abstract

This study explored the validity of an integrative framework for verbal and musical short-term memory (STM). Following this framework, access to domain-specific long-term knowledge bases supports the processing of musical and verbal item information in STM, while domain-general ordering processes support the representation of serial order information in the two domains. We exposed participants to verbal and musical STM tasks assessing either item information, order information, or both item and order information. Using an interindividual differences approach, we observed that performance in item-based STM tasks was not strongly associated between musical and verbal domains. In contrast, strong between-domain associations were observed for STM tasks assessing processing of verbal order and musical rhythm information. These preliminary results are overall in agreement with an integrative approach of verbal and musical STM. At the same time, the results highlight the difficulty of measuring serial order processing in the musical STM domain in a direct and specific manner.

## Introduction

Speech and music are both characterized by complex sound sequences that need to be maintained in short-term memory (STM) for further processing and comparison (Hickok, Buchsbaum, Humphries, & Muftuler, 2003; Janata, Tillmann, & Bharucha, 2002; Pfordresher, Palmer, & Jungers, 2007). While our understanding of the cognitive processes involved in the short-term maintenance of verbal memoranda has increased considerably over the last decades (for a recent review, see Majerus, 2013), we have very little knowledge about the mechanisms supporting short-term retention of musical material and, more critically, concerning the tasks most relevant for assessing the musical STM system. Building on verbal models of STM, we used an interindividual differences approach to explore the associations and dissociations between classical verbal STM tasks assessing item and/or order information retention capacity, and their musical counterparts developed for the purpose of the present study. Our goal was, in light of data available in the verbal STM literature, to explore the cognitive structure of musical STM through a direct comparison between musical and verbal STM tasks, allowing us to assess the level of commonalities and specificities between the two domains.

Numerous theoretical accounts have been developed in the field of verbal STM. A majority of these models assume that distinct processes support the short-term maintenance of memoranda in a sequence (i.e. item-related information) and the short-term retention of their order of occurrence (i.e. serial order information) (e.g., Brown, Preece, & Hulme, 2000; Burgess & Hitch, 2006; Lee & Estes, 1981; Majerus, 2013; Oberauer, Lewandowsky, Farrell, Jarrold, & Greaves, 2012). This is supported by data showing that psycholinguistic factors, such as lexicality, lexical frequency, phonotactic frequency or semantic relatedness, influence item but not serial order STM capacities (Gathercole, Frankish, Pickering, & Peaker, 1999; Hulme et al., 1997; Hulme, Maughan, & Brown, 1991; Majerus & Van der Linden, 2003; Poirier & Saint-Aubin, 1995, 1996; Roodenrys, Hulme, Lethbridge, Hinton, & Nimmo, 2002; Thorn, Gathercole, & Frankish, 2005). Serial order information is most often considered to be represented via independent serial positional markers associating items to specific positions in a sequence; these makers can take several forms such as dynamic temporal signals coding serial order at different temporal scales or signals representing the start and the end of a list (Brown et al., 2000; Burgess & Hitch, 2006; Hartley, Hurlstone, & Hitch, 2016; Henson, 1998). In addition, domain-general attentional processes are considered to support both item- and order-level maintenance processes, by focusing attention on memoranda and their order, and/or by allowing attentional refreshing of the stored information (Barrouillet, Bernardin, & Camos, 2004; Cowan, 1995). Considered together, these data are in line with theoretical models suggesting that the capacity to maintain verbal information over the short-term is an emergent property resulting from interactions between domain-specific long-term memory (LTM) linguistic knowledge and domain-general attention and serial ordering processes (Cowan, 1995; Majerus, 2013; Postle, 2006).

The same interactive principles may also be involved for the short-term maintenance of musical and verbal stimuli. However, we have very little knowledge about the structure and mechanisms that support STM for musical information. One of the few theoretical models for musical STM has been proposed by Berz (1995; see also Pechmann & Mohr, 1992). This model is based on the multicomponent model of working memory (Baddeley & Hitch, 1974), but adds a module specialized in the processing of musical stimuli which is furthermore considered to interact with musical LTM, although these interactions are not clearly specified. In another model of musical working memory, Ockelford (2007) proposed to add a musical executive system connected to the central executive component of the multicomponent model of working memory (Baddeley & Hitch, 1974). Ockelford (2007) also integrated short- and long-term musical stores, both being connected to the musical executive. However, these architectures remain fairly general and do not address the distinction between item and serial order aspects that are known to characterize verbal STM. Recent study suggests that this may also apply to the musical domain. Gorin, Kowialiewski, and Majerus (2016) showed that in musical and verbal STM tasks retention of serial order information, but not of item information is similarly impacted by a serial order interfering task, raising the possibility of domain-general serial order coding mechanisms. The present study will contribute to further explore this possibility.

Concerning the representation of item information in musical STM, the most basic unit of musical information is represented by tones, but the item level can also be represented by tone interval size. Consequently, item information in musical STM could be represented by both absolute pitch (the height of tones in a sequence) and relative pitch (the absolute interval size between consecutive tones in a sequence). Similar to what has been observed in the verbal domain, short-term recognition for tone sequences is influenced by musical knowledge stored in LTM, as reflected by improved musical short-term recognition capacities for tonally-organized sequences (Schulze, Dowling, & Tillmann, 2012) or for sequences composed of stimuli with familiar timbre (Siedenburg & McAdams, 2017), in line with the proposal of Berz (1995) and Ockelford (2007).

Even though little is known about the nature of serial order retention processes in musical STM, some studies suggest that serial order plays a critical role in tasks requiring the production of musical sequences (Pfordresher et al., 2007). These tasks are characterized by patterns of serial order errors which are similar to those observed in verbal STM tasks (Mathias, Pfordresher, & Palmer, 2015), such as transposition gradients, and transposition errors involving items of distant serial positions but sharing the same metrical signature which resemble interposition errors observed in the verbal domain (Henson, 1996). Also, in studies conducted on non-musician participants, we recently showed that musical STM for serial order is characterized by similar ordering effects as those witnessed in verbal STM tasks (Gorin, Mengal, & Majerus, 2018b, 2018a), as well as a by a similar sensitivity to timing-based interference (Gorin et al., 2016). This raises the question of the existence of cross-modal serial order STM mechanisms. This question of domain-general serial order STM mechanisms has also been raised for other STM domains such as visuo-spatial STM. Hurlstone, Hitch, and Baddeley (2014) recently argued for domain-general serial order STM mechanism across verbal and visuo-spatial domains, based on the observation of a large set of similar serial order phenomena across verbal and visuo-spatial STM tasks.

We have described here preliminary evidence suggesting that the serial order processes subserving the functioning of verbal STM could be extended to the musical domain (Gorin et al., 2016, 2018b, 2018a). We also reported that the dissociation between item and order processing characterizing several recent models of verbal STM could also be characteristic of musical STM (Gorin et al., 2016). These data suggest that the principles underlying the cognitive structure of verbal STM may also characterize musical STM. The goal of this study is thus to further our understanding of the structure of musical STM by exploring the associations between item and order STM tasks in the verbal and musical domains. We hypothesized the existence of domain-general ordering processes, but domain-specific item processing.

## The present study

The present exploratory study examined the extent to which verbal and musical STM rely on domain-specific processes, and this more specifically for item retention processes, and the extent to which verbal and musical STM rely on domain-general processes, and this more specifically for the retention of serial order information. To reach this aim, participants with low levels of musical expertise were exposed to different verbal and musical STM tasks, assessing either STM for item, STM for order information, or STM for both item and order information. We determined the extent to which performance on the different tasks was associated, using an interindividual differences perspective. This approach could have the potential to improve our understanding of the different specific and general components underlying verbal and musical STM capacities, and also further the development of tools for assessing musical STM in a precise and theoretically informed manner. Although this study was of exploratory nature, we hypothesized that cross-domain associations should be stronger for estimates of serial order STM as compared to estimates of item STM, considering that the order versus item dissociation observed in the verbal domain also applies to musical STM.

### Method

#### Participants

Ninety-five participants participated to the present experiment on a voluntary basis. Two participants had to be discarded due to technical problems during data acquisition. The data of 93 participants were retained for analysis (M_age_ = 22.0 years, SD = 2.5, range = 18 to 29; 60 women and 33 men). Musical expertise of participants was very low, with on average 1.3 years of singing or musical instrument practice (SD = 2.4; ranging from 0 to 12 years). All participants showed a high educational level, with on average 14.3 years of formal education (SD = 1.9; ranging from 10 to 21 years). No participant reported specific hearing impairment or having absolute pitch processing, except for one participant reporting episodes of mild tinnitus. The experiment had been approved by the local ethics committee and all the participants provided their written informed consent before starting the experiment.

#### Task overview

In the verbal domain, we assessed item STM via a single nonword delayed repetition task that maximizes the retention of sublexical phonological information while reducing serial order retention requirements (Gupta, 2003; Leclercq & Majerus, 2010; Majerus, Poncelet, Greffe, & Van der Linden, 2006). A similar item STM task was developed for the musical domain. We adapted the single-pitch and pitch-interval imitation tasks of the Sung Performance Battery (Berkowska & Dalla Bella, 2013), introducing a short-term delayed repetition procedure that imitated the procedure used for the nonword delayed repetition task. Note that, contrary to the verbal domain for which it has been established that repetition of short, monosyllabic nonwords is associated with phonological item memory (Attout, Van der Kaa, George, & Majerus, 2012; Gathercole, 1995; Majerus, 2013), there is, to the best of our knowledge, no agreement about the type of material (tones or tone intervals) that provides an optimal measure of item processing in musical STM tasks. Therefore we used both single tones and tone intervals for assessing item STM in the musical domain via an item delayed repetition task. A second set of tasks assessed the processing of item information in verbal and musical STM domains using recognition responses. This allowed us to determine whether a possible absence of associations between verbal and musical item STM tasks is due to intrinsic differences in verbal and musical STM capacities, or whether this absence could be better accounted for by the distinct response types characterizing the nonword repetition and tone reproduction tasks. Indeed, tone reproduction via singing responses may be an unfamiliar experience for non-musician participants and could lead to a biased estimate of musical item STM performance.

A next set of tasks assessed serial order STM capacities in verbal and musical domains. In the verbal domain, we used a serial order reconstruction task (see, e.g., Majerus, Poncelet, Van der Linden, & Weekes, 2008). In this task, item processing load is highly reduced by exposing participants to sequences of highly familiar items (digits), and by making the items available at recall, and hence only serial order information has to be maintained and reconstructed. Moreover, serial order reconstruction tasks are frequently used in experiments aiming at distinguishing between item and order processes in verbal STM (Attout et al., 2012; Brock & Jarrold, 2005; Majerus, Metz-Lutz, Van der Kaa, Van der Linden, & Poncelet, 2007). In the musical domain, we used the tone serial recall task developed by Williamson, Baddeley, and Hitch (2010). In that task, participants are exposed to musical sequences sampling tones from three pitch height categories, i.e. “low”, “medium”, and “high”. Participants are asked to reconstruct the order of occurrence of these three tone categories in a sequence by marking the tones on a visual grid in correct serial position. Serial order STM was also assessed via a rhythm recognition task from the Montreal Battery of Evaluation of Amusia (Peretz, Champod, & Hyde, 2003). This choice was motivated by the fact that previous studies evidenced a link between rhythm processing and memory for serial order (Gorin et al., 2016; Hartley et al., 2016; Henson, Hartley, Burgess, Hitch, & Flude, 2003; Plancher, Lévêque, Fanuel, Piquandet, & Tillmann, 2018; Saito, 2001). At the theoretical level, it has been argued that rhythm processing involves the same temporal oscillators as those that may also encode serial order information in STM tasks (Brown et al., 2000). We therefore considered rhythm processing to provide a further measure of the possible domain-general factor supporting serial order processing in STM. Furthermore, the rhythm STM task was selected as it clearly targets STM for temporal order information which is a major component of both auditory-verbal and musical processing. Most importantly, this task is a serial order recognition task as negative trials were always comprised of two adjacent tones (that differed in duration) whose temporal duration, but not absolute serial position was exchanged relative to the target sequence. Consequently, only the order of the duration of tones changed between the memory and recognition lists; the identity of the tones and their absolute positional order remained unchanged.

A further set of tasks were standard immediate serial recall tasks, which combine STM for item and serial order information. For the verbal domain, we confronted participants to an immediate serial recall task for word lists (Majerus & Van der Linden, 2003). For the musical domain, we used a hybrid recognition/recall task for item and order musical information developed by Gorin et al. (2016). This task uses a recall-like method, while avoiding biases related to singing performance as opposed to memory performance (see below for more details). The task was also chosen because it allows to break down performance into separate item and order scores. As shown by Gorin et al. (2016), an interference task targeting serial order processing has a selective impact on recognition for trials manipulating order information, but not on trials manipulating item information in this task.

Finally, musical perceptual capacities were assessed by two additional tasks involving discrimination of pitch and interval information. Table 1 displays a summary of all the tasks used, their STM domain, the STM component involved, as well as a short description of task requirements.

**Table 1:** Summary of the STM tasks used in the present experiment.

Task	Domain	STM component	Task description

Single nonword delayed repetition	Verbal STM	Item	Repetition of a single nonword after a delay filled with articulatory suppression
Nonword list recognition	Verbal STM	Item	Short-term recognition of nonword lists (manipulation of nonword identity only)
Single-pitch immediate repetition	Musical processing	NA	Reproduction of a single pitch
Pitch-interval immediate repetition	Musical processing	NA	Reproduction of a single pitch-interval (i.e., two consecutive pitches)
Single-pitch delayed repetition	Musical STM	Item	Reproduction of a single pitch after a delay filled with articulatory suppression
Pitch-interval delayed repetition	Musical STM	Item	Reproduction of a pitch-interval (i.e., two consecutive pitches) after a delay filled with articulatory suppression
Tone list recognition	Musical STM	Item	Short-term recognition for tone lists (manipulation of tone identity only)
Serial order reconstruction for digits	Verbal STM	Order	Immediate serial reconstruction of digit lists of length 6 to 9
Serial order reconstruction for tones	Musical STM	Order	Immediate serial reconstruction of tone lists (three pitch categories) of length 3 to 7
Short-term memory for rhythm	Musical STM	Order for rhythm	Short-term recognition of melodies (manipulation of rhythmic component only)
Word list immediate serial recall	Verbal STM	Item and order	Immediate serial recall of word lists of length 5 to 7
Tone list immediate serial recognition	Musical STM	Item and order	Serial order recognition for tones

#### Material, procedure and scoring

##### Short-term memory for item information

*Single nonword delayed repetition.* The stimuli used for this task consisted in a set of 12 nonwords with a CVC syllabic structure. The nonwords were legal as regards the French phonotactic rules and the CV and VC diphones had a low phonotactic frequency according to Tubach and Boë (1990) (CV segments: M = 205, SD = 132, range = 27 to 419; VC segments: M = 144, SD = 215, range = 7 to 589). The nonwords had been recorded by a French-speaking male speaker (mean duration of 639 milliseconds, SD = 90). Note that we used nonwords of low phonotactic frequency in order to maximize the assessment of phonological item memory while reducing the contribution of long-term phonological knowledge. Also, in previous studies in children using similar CVC nonword stimuli for nonword repetition tasks, phoneme-level serial order errors occurred very rarely (see, Leclercq & Majerus, 2010). The nonwords were presented at a comfortable output level through headphones connected to a portable workstation. Immediately after presentation, participants were required to repeat the nonword once (in order to ensure that the nonword had been correctly encoded) and immediately after started counting backwards from 99 by steps of two for a period of seven seconds in order to prevent the refreshing of the nonword item via subvocal articulatory rehearsal. After the 7-second period, a blue circle appeared at the center of the screen, requiring participants to recall the nonword. At the end of each trial, participants pressed a key to start the next trial. The responses were recorded for later transcription and scoring. There were 24 experimental trials preceded by two practice trials composed of nonwords not used in the experimental trials. Each of the 12 nonwords was presented twice over the experiment; in order to match the structure of the corresponding musical task. The trials were presented in a pre-established pseudo-random order (the same nonword could not occur on two successive trials) via Opensesame software (Mathôt, Schreij, & Theeuwes, 2012). A response was scored as correct when the delayed repetition of the nonword matched its immediate repetition, thereby ruling out that erroneous responses could result from misperceptions rather than from memory problems (for a similar scoring method, see Leclercq & Majerus, 2010).

*Single-pitch and pitch-interval delayed repetition.* The task was an adaptation of the single-pitch and pitch-interval matching tasks developed for the Sung Performance Battery (Berkowska & Dalla Bella, 2013). The stimuli were 1-second sine-wave tones. For single-pitch delayed repetition, the stimuli consisted in the 12 steps of a chromatic scale centered on the vocal range of the participants. For delayed pitch-interval repetition, the same tones were arranged in ascending and descending pairs, resulting in 26 intervals from a minor second to an octave, including two unison intervals (0 semitones interval). The vocal range was determined by asking participants to produce two glissandi from low to high and high to low pitch, as in Larrouy-Maestri and Morsomme (2014). We used the mean of the two glissandi to center the stimuli on participants’ vocal range. For the single-pitch repetition task, there were four practice trials and 24 experimental trials; the 12 tones were presented twice during the task. For the delayed pitch-interval repetition task, participants were exposed to four practice trials followed by 26 experimental trials composed of 12 ascending, 12 descending and 2 unison intervals. The single-pitch repetition task always preceded the pitch-interval repetition task, with a short break between the two. For each task, after its initial repetition, participants repeated the tone/interval after a 7-second delay filled with a backwards counting task as for the nonword delayed repetition task. As verbal and musical articulatory suppression have a similar effect on verbal and musical STM tasks (Schendel & Palmer, 2007), we used the same type of verbal articulatory suppression in the nonword repetition and the two musical repetition tasks. For the single-pitch repetition task, reproduction accuracy was expressed as the deviation in cents (100 cents = 1 semitone) of the recalled pitch relative to its immediate repetition. For the pitch-interval repetition task, we determined the interval size in cents of the recalled and immediately produced intervals, and computed the absolute difference between them (for similar scoring methods, see Pfordresher & Brown, 2007). As for the nonword delayed repetition task, a pseudo-random stimulus presentation order was chosen, ensuring that the same tone could not occur on two successive trials. The task was presented via Opensesame (Mathôt et al., 2012), and the tone/interval productions were recorded for later analysis and scoring. As in the single nonword delayed repetition task, musical STM for item information was assessed, for the two tasks, by computing the absolute deviation between the delayed repetition and the immediate repetition to reduce the influence of singing abilities on the score thought to reflect musical STM for item. We also used the score from the immediate repetition in the two tasks to assess and control for singing abilities in subsequent analyses.

*Nonword list recognition.* The stimuli used for this task were 10 nonwords following a CVC syllabic structure. The nonwords were different from those used in the single nonword delayed repetition task, and were legal regarding to French phonotactic rules. Phonotactic frequency of CV and VC diphones was again low (CV segments: M = 213, SD = 99, range = 60 to 314; VC segments: M = 171, SD = 201, range = 29 to 543). The nonwords had a mean duration of 725 milliseconds (SD = 102) and had been recorded by a French-speaking male speaker. The task was composed of 56 experimental trials and four training trials (one for each of the four list lengths). For training trials, participants received feedback in order to ensure familiarization with the task procedure. The trials consisted of nonword lists of increasing length (from two to five), with a number of trials corresponding to their list length multiplied by four (length 2: eight trials; length 3: 12 trials; length 4: 16 trials; length 5: 20 trials). This was done to ensure that each serial position was probed twice for each list length. The nonwords were presented at the rate of one nonword per second. Pairs of nonword lists (target and probe lists) were presented at a comfortable level through headphones connected to a portable workstation. The two lists of a pair were separated by a 3-second maintenance phase. After the 3-second period and the presentation of the probe list, participants made a same-different judgment by pressing the corresponding response button. Half of the trials were matching trials and the other half were non-matching trials in which one of the probe nonwords differed from its target by only its initial consonant; furthermore the initial consonant of the probe word was not shared by any other nonword of the probe list. We also ensured that all the trials were unique. The trials were presented in a pre-established pseudo-random order via Opensesame software (Mathôt et al., 2012). Mean response accuracy over the 56 experimental trials was determined.

*Tone list recognition.* This task was similar to the nonword list recognition tasks while assessing short-term recognition of tone identity. The material consisted in a set of six 1-second sine-wave tones (C_5_, D_5_, D^#^_5_, F^#^_5_, G^#^_5_, and A_5_) generated by the program controlling the task. The procedure was exactly the same as for the nonword list recognition task and also contained the same number of trials and sequence lengths. For different trials, mismatching sequences were constructed by replacing a tone from the target sequence by a tone one semitone lower or higher than the target tone. As unfamiliar nonwords were used for the verbal counterpart of this task, we assessed the tonal strength of the tone sequences based on the Krumhansl and Schmuckler algorithm (cited in Krumhansl, 1990) to ensure they did not induce a familiar tonal context. The algorithm correlates the distribution profile of pitch class occurrences of the target sequences with the distribution profiles of the 12 major and 12 minor key tones profiles developed by Krumhansl and Kessler (1982). We observed only small correlations with the different key tone profiles. We report here the four highest correlations that were observed: *r* =.14 with D/G^#^ major scale for 2-tone lists, *r* = 0.29 with D^#^ minor for 3-tone lists, *r* = 0.24 with F^#^ minor for 4-tone lists, and *r* = 0.30 with F^#^ minor for 5-tone lists. Task administration was controlled via Opensesame (Mathôt et al., 2012) and we determined the mean proportion of correct response over the 56 trials.

##### Short-term memory for serial order

*Serial order reconstruction for digits.* The task was adapted from the serial order reconstruction task used by (Majerus et al., 2008). The stimuli consisted in a pool of nine spoken digits (from 1 to 9) recorded by a French-speaking male speaker (mean duration = 411 milliseconds, SD = 174). The task was composed of 16 experimental trials and four practice trials. The experimental trials were digit lists of increasing length (from 6- to 9-digit lists), with four trials for each length condition; the practice trials included 5 digits. The digits used were sampled from 1 to *N, N* corresponding to sequence length (e.g., for list length 6 the digits used were the digits 1 to 6). This procedure allows maximizing serial order processing requirements by ensuring that item information is known in advance. The digit lists were presented by increasing length at a comfortable output level through headphones connected to a portable workstation and the lists were played at the rate of one digit per second. Directly after the presentation of each digit list, participants were required to reconstruct the order of occurrence of the digits presented in the trial by using cards on which the digits presented in the sequence were printed. The participants had to arrange the cards following the order of occurrence of the digits in the STM list. Before each trial, the cards were arranged horizontally in numerical order on the desk and hidden by a mask; the mask was removed immediately after the presentation of the last item of each sequence. When participants completed a trial, they pressed a button to advance to the next trial. Participants were informed when sequence length increased by a message appearing on the computer screen. We determined the proportion of digits placed in correct serial position over all the trials.

*Serial order reconstruction for tones.* Similar task was created for serial order reconstruction of tone sequences and was inspired by a task developed by Williamson et al. (2010). The stimuli used for this task were three 800-millisecond sine-wave tones of 524, 784 and 988 Hertz, corresponding respectively to C_5_, G_5_ and B_5_. As in Williamson et al. (2010), participants first passively listened to 10 successive presentations of the three different tones played in ascending order (from C_5_ to B_5_). This was done to ensure that the participants were familiarized with the tones used in the task. Next, participants performed the 20 experimental trials and five practice trials involving tone sequences of increasing length (from 3- to 7-tone lists; four trials per length and one practice trial). The tones were played at the rate of one tone per second. Each of the three tones occurred equally often over all trials and serial positions, all adjacent tones in a given trial were different, and each sequence was unique. We also ensure that the three tones could not appear in a simple ascending or descending order (e.g., C_5_–G_5_–B_5_ or B_5_–G_5_–C_5_). Participants were required to listen to the sequences presented at a comfortable output level through headphones. After each sequence, participant were asked to reconstruct the order of the tones by marking by hand with a pencil the order of the tones in a visual grid as in Williamson et al. (2010). The visual grid consisted in a 3-row (“low”, “medium”, and “high” pitch height categories) by *n*-columns matrix, *n* corresponding to the number of tones in a sequence, and was handed to participants after each sequence presentation. When participants completed a trial, they pressed a button to advance to the next trial; participants were informed when sequence length increased by a message appearing on the computer screen. The trials were presented in a pre-established pseudo-random order via Opensesame software (Mathôt et al., 2012). The proportion of tones correctly reconstructed over all the trials was determined.

*Short-term memory for rhythm.* In order to assess STM capacities for rhythmically organized musical information we used the rhythm subtest of the Montreal Battery of Evaluation of Amusia (Peretz et al., 2003). This task requires participants to determine if pairs of melodies are same or different, different trials presenting sequences composed of the same melodic content but with a different rhythmical structure by exchanging the duration of two adjacent tones between the target and probe sequences. The stimuli used were the audio files provided by Peretz et al. (2003) and we followed the procedure described by the authors. The target sequences where of different tone length, lasting between 3.8 and 6.4 seconds (mean: 5.1 seconds), and target and comparison sequences were separated by a 2-second silent interval. The proportion of correct responses over the 30 experimental trials was determined.

##### Short-term memory for item and order information

*Word list immediate serial recall.* We used an immediate serial recall task for lists of frequent words developed by Majerus and Van der Linden (2003). The stimuli were 108 frequent bisyllabic words coming from an open set, with a frequency count higher than 10 000 (Content, Mousty, & Radeau, 1990). The task consisted of 24 word-lists of increasing length (2 to 7 words), with four trials for each list length. The order of presentation of the lists was the same as in Majerus and Van der Linden (2003) and was controlled by the software Opensesame (Mathôt et al., 2012), and each word occurred only once over all the trials. The lists had been recorded as a unique. wav files by a French-speaking male speaker, at the rate of one item per second. The lists were presented via headphones connected to a portable workstation and by increasing length for immediate serial recall. Participants were asked to say “forgotten” if they did not remember the word for a given serial position. Responses were recorded for later scoring. After each trial, the participants pressed a button to start the next trial; participants were informed when list length increased. We determined the proportion of items recalled in correct serial position (reflecting thus both item and order information). We also computed an item score corresponding to the proportion of items correctly recalled independently of their serial position, as well as an order score corresponding to the proportion of items recalled at their correct serial position relative to the items recalled independently of their position. We only retained for analysis performance on trial lengths 5 to 7 in order to match the sequence length used in the musical version of the task, and due to ceiling effects for list lengths 2 (M > 0.99, SD = 0.02), 3 (M = 1.00, SD = 0.00), and 4 (M = 0.95, SD = 0.10).

*Tone list immediate serial recognition.* This task was designed to measure item and order retention for musical stimuli and was adapted from Gorin et al. (2016). The stimuli used were eight 800-millisecond sine-wave tones corresponding to all the steps of a C major scale beginning with C_4_ (264 hertz) and ending with C_5_ (523 Hertz). The experiment consisted of 72 experimental trials (and 6 practice trials) presenting tone sequences of increasing length (from 5- to 7-tone lists), with tones presented at the rate of one per second. In order to ensure that for each list length each serial position was probed four times, the number of trials per list length was different (length 5: 20 trials; length 6: 24 trials; length 7: 28 trials). The tone sequences induced a familiar tonal context of C major, in order to parallel the familiar word stimuli used for the verbal equivalent of this task (Krumhansl & Kessler maximum correlation with C major key for length 5: M = 0.73, SD = 0.06, for length 6: M = 0.73, SD = 0.10, and for length 7: M = 0.75, SD = 0.09). The sequences were presented at a comfortable output level. The tones of the sequences were presented in time with a beat serving as a metronome. After a 3-second silent period, a circle flashed two times on the center of the screen and the metronome was started again; participants had to covertly recall the tone sequence in time with the metronome while a probe tone was presented at one serial position. After hearing the probe, participants had to decide if the probe tone matched the tone at the same serial position in the target sequence by pressing one of the two corresponding response keys. Half of the trials were matching trials. For mismatching trials, half of the trials involved tones not presented in the target sequence (item mismatch) and the other half involved tones of the target sequence but presented in a wrong serial position (serial order mismatch). Participants were instructed to decide whether the probe tone matched both item and order information; if one of the information types was mismatching, they had to give a no response. After the participant’s response, the next trial started automatically; participants were advised when sequence length increased. The trials were presented in a pre-determined pseudo-random order via Opensesame software (Mathôt et al., 2012) and we ensured that each sequence was unique. In addition to the overall recognition accuracy score, we determined an item score based on the rate of correct responses for item-based mismatching trials, and an order score based on the rate of correct response to order-based mismatching trials.

*Musical perception and discrimination.* In order to assess musical discrimination abilities we used the pitch and interval discrimination tasks used in Pfordresher and Brown (2009). The stimuli were 1-second sine-wave tones generated directly by the Opensesame software which also controlled task presentation (Mathôt et al., 2012). For the pitch discrimination task, participants were exposed to 24 experimental trials (and four practice trials). The trials consisted in pairs of single pitches separated by a 1-second silent period. The pitch target was always C_5_ (523 Hertz), followed either by an identical comparison pitch (50% of the trials) or by a different comparison pitch (50% of the trials). Non-matching stimuli differed from the target tone by 25, 50, 100, 200, 400, 600 or 800 cents, in either direction. Participants made same-different judgments using two response keys. The proportion of correct responses over the 24 experimental trials was determined. For the interval discrimination tasks, 20 experimental trials (and four practice trials) involved the presentation of two tone pairs separated by a 1-second pause. The target interval (first tone pair) consisted of the tone C_5_ followed by G_5_ (523 Hertz and 784 Hertz, respectively), corresponding to a rising interval of 700 cents. The comparison intervals always began with F^#^_5_ (740 Hertz). In 50% of the trials, F^#^_5_ was followed by C#_6_ (1109 Hertz), thus forming a 700-cent rising interval identical to the target interval. In the other trials, F^#^_5_ was followed by a tone smaller or higher by 25, 50, 100, 200, or 400 cents than C#_6_. Participants made a same-different judgment for the two interval sizes by using two response keys. Response accuracy over all the experimental trials was determined.

#### Task order

The 10 tasks used in this experiment were spread into two blocks of five tasks, each lasting approximately one hour and half, by alternating between verbal and musical tasks. The first block contained, in successive order of administration, the tone list immediate serial recognition task, the digit serial order reconstruction task, the tone list recognition task, the single nonword delayed repetition task, and the musical discrimination task. The second block presented, from the first to last task, the rhythm STM task, the word list immediate serial recall task, the tone serial order reconstruction task, the nonword list recognition task, and the single-pitch and pitch-interval delayed repetition tasks. The order of the presentation of the tasks was fixed within each block, but the order of presentation of the two blocks was counterbalanced across participants.

#### Statistical analyses

All analyses used a Bayesian statistical framework. We used JASP software using default settings (JASP Team, 2018, version 0.8.0.0), as well as the *BayesFactor* package (Morey & Rouder, 2015, version 0.9.12-2) ran in *R* (R Core Team, 2014). For Bayesian regression, we used the *regressionBF* function comparing simultaneously several models (combinations of predictor variables) relative to the null model (model with only the intercept). The specificity of effects was determined using the “top” argument of the “whichModels” parameter in the *regressionBF* function. This method allows to test the specific effect of a covariate by comparing the evidence for the most complex model containing all the covariates relative to the same model without the covariate of interest (Rouder & Morey, 2012). When the full model is preferred over the model without the covariate, this represents evidence for the contribution of the covariate in explaining the data.

In the following *Results* section, we report three types of analyses. After an initial set of descriptive analyses exploring the sensitivity of the different measures (see Table 2), a second set of exploratory correlation analyses assessed the patterns of associations between the different tasks which were designed to assess only one STM component (item or order). One has to note that correlations conducted between STM tasks assessing the item component also included the immediate imitation component of the single-pitch and pitch-interval repetition tasks which assessed singing accuracy. Based on the observed pattern of correlations between the different tasks, we decided to regroup the tasks as a function of verbal item, musical item, and domain-general serial order STM components (see the *Results* section for more details). Note that the formation of a domain-general order component is also supported by empirical evidence supporting the view that the processing of serial order information in verbal and musical domains of STM involves similar mechanisms (Gorin et al., 2016, 2018b, 2018a). The construction of the composite scores was based on a data-driven approach and was done in order to reduce the complexity of the subsequent set of regression analyses. These regression analyses aimed at determining the extent to which performance in verbal and musical STM tasks assessing both item and order components are predicted by domain-specific item and domain-general serial order STM components from the two auditory domains.

**Table 2:** Descriptive statistics for the different experimental tasks.

Task	Mean	Median	SD	Min.	Max.

*Verbal item*					
Single nonword delayed repetition (Proportion of nonwords correctly recalled; *n* = 24)	0.85	0.88	0.12	0.50	1.00
Nonword lists recognition (Proportion of correct recognitions; *n* = 56)	0.71	0.70	0.08	0.52	0.91
*Musical item*					
Single-pitch immediate repetition* (mean absolute deviation; *n* = 24)	195 [140]	159 [88]	132 [143]	20 [2]	486 [522]
Pitch-interval immediate repetition* (mean absolute deviation; *n* = 26)	162 [127]	142 [62]	101 [164]	27 [6]	554 [646]
Single-pitch delayed repetition* (mean absolute deviation; *n* = 24)	117	96	78	13	356
Pitch-interval delayed repetition* (mean absolute deviation; *n* = 26)	199	138	172	23	979
Tone lists recognition (Proportion of correct recognitions; *n* = 56)	0.72	0.73	0.12	0.48	0.98
*Verbal order*					
Serial order reconstruction for digits (Proportion of digits recalled at the correct position over all the trials; *n* = 120)	0.77	0.78	0.11	0.50	1.00
*Musical order*					
Serial order reconstruction for tones (Proportion of tones recalled at the correct position over all the trials; *n* = 100)	0.71	0.74	0.16	0.29	0.99
Short-term memory for rhythm (Proportion of correct recognitions; *n* = 30)	0.85	0.87	0.09	0.53	1.00
*Word list immediate serial recall*					
Global score (Proportion of words recalled at the correct position across 5- to 7-list length trials; *n* = 72)	0.72	0.72	0.10	0.50	0.99
Item score (Proportion of words recalled independently of their position across 5- to 7-list length trials; *n* = 72)	0.82	0.82	0.07	0.61	0.99
Order score (The ratio between the global score and the item score)	0.87	0.88	0.07	0.67	1.00
*Tone list immediate serial recognition*					
Global score (Proportion of correct recognitions over all the trials; *n* = 72)	0.67	0.68	0.09	0.43	0.96
Item score (Proportion of correct recognitions over all item-related different trials; *n* = 18)	0.58	0.56	0.15	0.22	1.00
Order score (Proportion of correct recognitions over all order-related different trials; *n* = 18)	0.60	0.61	0.18	0.11	1.00
*Musical discrimination*					
Pitch discrimination (Proportion of correct responses; *n* = 24)	0.93	0.93	0.05	0.82	1.00
Interval discrimination (Proportion of correct responses; *n* = 20)	0.63	0.65	0.15	0.30	0.90

Scores into brackets are norms for immediate imitation of single-pitch and pitch-interval tasks developed by Berkowska and Dalla Bella (2013). Legend: * scores are expressed in cents (100 cents = one semitone).

## Results

### Descriptive analyses

Descriptive statistics for the 93 participants included in this study are provided in Table 2. Tasks were characterized neither by ceiling or floor effects. For tasks involving a same/different judgement, the lowest performance was observed for the interval discrimination task with a mean of 0.63 (SD = 0.15), which is still higher than the 0.50 chance-level. The highest performance was observed for the pitch discrimination task with a mean of 0.93 (SD = 0.05), indicating that pitch discrimination was much easier than interval discrimination in our participants. Finally, concerning immediate singing tasks, deviation rates are in line with those reported in Berkowska and Dalla Bella (2013), even though our results indicate that our sample and/or task may have led to slightly less accurate reproduction than reported in Berkowska and Dalla Bella (2013). In their study, participants heard the to-be-imitated target twice and had to produce two imitations of each target, which may have contributed to the slightly higher accuracies.

### Correlation analyses

#### Item short-term memory measures

As shown in Table 3, there were, as expected, no reliable associations between verbal and musical STM tasks that maximized retention of item information. The correlations between the single nonword delayed repetition task and the different musical item STM tasks were characterized by low BF_10_ values, ranging from 0.26 to 0.33, and corresponding to BF_01_ values ranging from 3 to 3.85 and representing moderate evidence for an absence of association (see Table 3). A very similar pattern of results was observed for the association between the nonword list recognition task and the different musical item STM tasks (see Table 3), with BF_10_ values ranging between 0.42 and 0.19, corresponding to BF_01_ of 2.43 to 5.26, respectively. At the same time, a strong intra-domain correlation was observed between single nonword delayed repetition task and the nonword list recognition task (BF_10_ = 19.47). Given this strong correlation and in order to reduce the complexity of the regression analyses reported in the next section, we created a *verbal item composite score* by transforming raw scores into *z*-scores and averaging the *z*-scores over the two tasks. In the musical domain, strong correlations were also observed between the different musical item STM tasks (see correlations between tasks 3 to 5 in Table 3). As for the verbal domain, we created a *musical item composite score* by averaging *z*-score transformed performances over the tone list recognition, pitch-interval delayed repetition, and single-pitch delayed repetition tasks, after having inversed the sign of the scores for the two repetition tasks in order to harmonize the directionality of the scores between the different tasks. Interestingly, the results also provided decisive evidence for the existence of an association between performance in the tone list recognition task—that do not require singing—and immediate imitation performance for the single-pitch (BF_10_ = 151.46, *r* = –0.38) and the pitch-interval (BF_10_ = 6.84E+4, *r* = –0.51) production tasks, which assessed singing accuracy without a memory component. We should note that the pitch and interval discrimination tasks, and the single-pitch and pitch-interval immediate imitation tasks, were not included in the musical item composite score, Indeed, these two tasks are measures of sound discrimination abilities and singing accuracy, respectively, rather than musical STM.

**Table 3:** Raw correlations between short-term memory tasks for verbal and musical item, tasks assessing singing abilities and tasks for verbal and musical order, as well as corresponding BF statistical values.

Domain	Task		Verbal item		Musical item			Singing		Verbal order	Musicalorder	

		Measure	1	2	3	4	5	6	7	8	9	10

Verbal item	1. Single nonword delayed repetition	Pearson’s r	–	**0.33**	–0.12	–0.14	0.12	0.11	–0.08	**0.34**	0.03	0.08
		BF_10_	–	**19.47**	0.26	0.33	0.26	0.22	0.17	**37.04**	0.14	0.18
	2. Nonword list recognition	Pearson’s r		–	–0.16	–0.15	0.09	–0.09	–0.12	**0.41**	0.17	**0.36**
		BF_10_		–	0.42	0.37	0.19	0.19	0.25	**477.37**	0.45	**72.43**

Musical item	3. Single-pitch delayed repetition	Pearson’s r			–	**0.39**	**–0.42**	0.26	**0.36**	–0.19	–0.18	–0.19
		BF_10_			–	**202.74**	**830.06**	3.19	**55.71**	0.68	0.54	0.72
	4. Pitch-interval delayed repetition	Pearson’s r				–	**–0.45**	**0.30**	**0.46**	–0.29	–0.23	–0.19
		BF_10_				–	**3553.75**	**10.05**	**4503.03**	5.94	1.60	0.62
	5. Tone list recognition	Pearson’s r					–	**–0.38**	**–0.51**	**0.33**	**0.34**	0.29
		BF_10_					–	**151.46**	**6.84E+4**	**21.24**	**27.66**	6.02

Singing	6. Single-pitch immediate repetition	Pearson’s r						–	**0.67**	–0.07	–0.18	–0.20
		BF_10_						–	**3.01E+10**	0.16	0.60	0.81
	7. Pitch-interval immediate repetition	Pearson’s r							–	–0.21	–0.27	–0.29
		BF_10_							–	0.89	3.64	7.14

Verbal order	8. Serial order reconstruction for digits	Pearson’s r								–	0.28	**0.38**
		BF_10_								–	5.33	**130.99**

Musical order	9. Serial order reconstruction for tones	Pearson’s r									–	0.24
		BF_10_									–	1.90
	10. Short-term memory for rhythm	Pearson’s r										–
		BF_10_										–

Legend: For correlations supported by a BF_10_ larger than 10, Pearson’s *r* and associated BF are represented in bold.

#### Order short-term memory measures

The correlations between the STM tasks for serial and temporal order information (in musical and verbal domains) are shown in Table 4 (tasks 8 to 10). As suspected, the digit serial order reconstruction and the rhythm STM tasks showed a strong correlation (BF_10_ = 130.99). The correlation between the digit serial order reconstruction and the tone serial order reconstruction tasks was supported by moderate evidence (BF_10_ = 5.33). The link between the tone serial order reconstruction task and the rhythm STM task was also smaller and associated only with anecdotal evidence (BF_10_ = 1.90). Given little evidence for an association between the tone serial order reconstruction task and the two other serial order task, we created a *domain-general serial order composite score* by averaging *z*-scores for the most strongly associated variables, i.e. the digit serial order reconstruction and the rhythm STM tasks.

**Table 4:** Results of the top-down Bayesian regression analysis for the tone list immediate serial recognition task.

Predictor of interest	BF_10_	BF_01_

*Dependant variable: Global recognition accuracy*		
Pitch discrimination	0.33	3.01
Interval discrimination	0.32	3.13
Pitch imitation	0.39	2.54
Interval imitation	0.34	2.97
Domain-general serial order composite score	2.56	0.39
Verbal item composite score	1.04	0.96
Musical item composite score	25.0	0.04
*Dependent variable: Item change recognition accuracy*		
Pitch discrimination	0.56	1.77
Interval discrimination	0.625	1.60
Pitch imitation	1.51	0.66
Interval imitation	0.32	3.10
Domain-general serial order composite score	1.51	0.66
Verbal item composite score	0.51	1.97
Musical item composite score	33.33	0.03
*Dependent variable: Serial order change recognition accuracy*		
Pitch discrimination	0.40	2.47
Interval discrimination	0.34	2.93
Pitch imitation	0.49	2.02
Interval imitation	0.35	2.88
Domain-general serial order composite score	20.00	0.05
Verbal item composite score	0.72	1.38
Musical item composite score	1.45	0.69

Legend: BF_10_ relates the evidence for the full model relative to the same model without the predictor of interest (i.e. the evidence in favor of the predictor of interest) while BF_01_ represents the evidence for the model without the predictor of interest relative to the full model (i.e. the evidence against the predictor of interest).

### Regression analyses

Next, we conducted Bayesian multiple regression analyses in order to determine the extent to which the different composite scores predict performance in the combined item and order STM tasks. As single-pitch and pitch-interval immediate repetition tasks, as well as pitch and interval discrimination tasks were not included in any composite scores, these tasks were included in the multiple regressions analysis as control variables. We expected that verbal (nonword delayed repetition and nonword list recognition tasks) and musical (single-pitch and pitch-interval delayed repetition and tone list recognition tasks) item composite scores contribute to word list immediate serial recall performance and to tone list immediate serial recognition, respectively. Furthermore, the domain-general serial order composite score (digit serial order reconstruction and rhythm STM tasks) should be associated with the verbal and musical immediate serial recall/recognition tasks. More precisely, it was expected that the musical item and the domain-general serial order composite scores should contribute strongly to the item and order scores, respectively. For the tone list immediate serial recognition task, we predicted that the musical item and domain-general serial order composite scores should contribute more strongly to the item and order scores of the task. We also expected that, for the word list immediate serial recall task, the verbal item and domain-general serial order composite scores should contribute more strongly to item and order recall accuracy, respectively.

#### Word list immediate serial recall

We observed that the regression model explaining best the global score—requiring to recall the correct item at the correct position—was the model composed of only the domain-general serial order composite score (BF_10_ = 1.75E+8), favored by a ratio of 6.12 over the second best model which also included the musical item composite score (BF_10_ = 2.86E+7). The results therefore suggest than only the domain-general serial order composite score contributes in explaining performance in word list immediate serial recall. The same results were observed when the more specific item or serial order scores were considered as dependent variable, the model explaining the data best being each time the model containing only the domain-general serial order composite score (BF_10_ = 1.26E+10 and BF_10_ = 3.75E+4 for the item and serial order scores, respectively), this model being favored over the second best model by a factor of 6.06 and 4.46 when considering the item and order scores, respectively.

#### Tone list immediate serial recognition task

For the tone list immediate serial recognition task, we first analyzed predictors of the global score corresponding to recognition accuracy over all trials in this task. The model receiving the strongest evidence was the model with both the musical item and domain-general serial order composite scores as predictors (BF_10_ = 9.49E+6), this model being favored by a ratio of 1.67 over the same model including also the verbal item composite score (BF_10_ = 5.68E+6). In order to determine more precisely the contribution of each predictor separately, we compared the full model to the same model without the predictor of interest. As shown in Table 4, there was strong evidence for an effect of the musical item composite score (BF_10_ = 25.00); the other predictors received ambiguous evidence regarding their contribution to the global score (see the *Global score* section of Table 4 for BF values associated to each predictor). We next conducted the same analyses for the item recognition score, corresponding to performance on trials including an item change between the target tone list and the probe list. The model containing both musical item and domain-general serial order composite scores as well as the immediate single-pitch imitation tasks received the highest level of evidence (BF_10_ = 2.89E+6), this model being favored by a ratio of 1.90 over the second best model which is the same model but without the immediate single-pitch imitation task. When testing separately the contribution of each predictor we obtained decisive evidence for a contributing effect of the musical item composite score (BF_10_ = 33.33); ambiguous evidence was associated to the other predictors regarding their contribution to the item score (see the *Item score* part of Table 4 for BF values associated to each predictor). Finally, we analyzed the order recognition score corresponding to performance on trials including a serial position change of items in the target tone list and the probe list. When predicting the order recognition score, the model containing both musical item and domain-general serial order composite scores received the strongest evidence (BF_10_ = 1.15E+6). This model was favored by a factor of 2.48 over the second best model corresponding to the same model plus the verbal item composite score. Separate analyses of the effect of each predictor variable showed strong evidence for a contribution only of the domain-general serial order composite score (BF_10_ = 20.00); the other predictor received ambiguous evidence as regards their contribution to the order score (see the *Order score* part of Table 4 for BF values associated to each predictor).

To summarize, regression analyses conducted on the tone list immediate serial recognition task showed that the two main predictors of performance were the musical item and domain-general serial order composite scores. Importantly, when considering response accuracy only for different trials containing item changes, it appeared that the musical item, but not the order composite score was a specific predictor. In contrast, the domain-general serial order composite score was the only specific contributor to recognition accuracy for serial position changes.

## Discussion

In the present study, we observed evidence for an absence of association between performance in verbal and musical item STM tasks. On the other hand, strong associations were observed between verbal serial order STM and musical rhythm STM tasks. Furthermore, regression analyses showed that musical item and domain-general serial order composite scores predict performance in a musical immediate serial recognition task considered to measure both item and order retention capacities. Also, the musical item and domain-general serial order composite scores were specific predictors of item and serial order recognition performance, respectively, in the tone list immediate serial recognition task, further suggesting the dissociation of the two components in musical STM. At the same time, for the verbal STM task assessing both item and serial order retention capacities, performance was predicted by the domain-general serial order composite score but not by the verbal item STM composite score. The results partially support our expectations, but also reveal unexpected findings such as the link between singing abilities and musical memory or low degree of association between performance on a musical serial order reconstruction task and performance on both verbal serial order STM and rhythmic STM tasks. Similarly, the absence of prediction of verbal immediate serial recall performance by the verbal item STM composite score is not in line with our expectations. After discussing first the more general theoretical implications of our findings, we will next examine the unexpected findings.

### What cognitive structure for musical and verbal STM?

In sum, our results are in line with a theoretical position considering that in musical and verbal STM tasks, the identity of item information is processed and maintained via distinct representational stores, while the maintenance of sequential aspects is driven, at least partially, by shared serial-temporal mechanisms. This position reconciles the two theoretical accounts that have been proposed, one account considering that musical and verbal STM capacities are underpinned by fully independent cognitive systems (Berz, 1995; Deutsch, 1970; Ockelford, 2007; Pechmann & Mohr, 1992), and another account considering that verbal and musical STM systems present some overlap (Salamé & Baddeley, 1989; Siedenburg, Mativetsky, & McAdams, 2016; Tillmann, Lévêque, Fornoni, Albouy, & Caclin, 2016; Williamson et al., 2010). The results of our study suggest that the this overlap is due mainly to common processes involved in the representation of serial order information, while item information is processed by domain-specific systems also involved in the sensory processing of the corresponding information (see Patel, 2012, and Williamson et al., 2010, for related proposals).

Concerning the serial order STM measures, we observed particularly strong associations between the verbal serial order STM measure and the rhythm STM task. This result is in line with several studies showing the importance of timing and rhythm in processing serial order information in verbal STM (e.g., Gorin et al., 2016; Plancher et al., 2018; Saito, 2001). In the verbal STM field, numerous computational accounts of serial order STM have been proposed (see, e.g., Brown et al., 2000; Burgess & Hitch, 1992; Farrell & Lewandowsky, 2002; Hartley et al., 2016; Henson, 1998; Lewandowsky & Farrell, 2008; Lewandowsky & Murdock, 1989; Page & Norris, 1998). Some of these models rely strongly on the assumption that the codes representing serial order information are based on timing-sensitive signals or temporal components (Brown et al., 2000; Burgess & Hitch, 2006; Hartley et al., 2016). For instance, Hartley et al. (2016) suggested that serial order and its rhythmic structure in verbal STM can be represented through a bottom-up multi-scale population oscillator responding to local changes in the speech envelope. There is also growing evidence in the field of STM showing that the serial ordering processes reported in the verbal domain can be extended to the visuo-spatial domain (for a recent review, see Hurlstone et al., 2014) and the musical domain (Gorin et al., 2016), arguing in favor of the existence of domain-general serial order processes in STM. However, since in the present study a reliable link was observed only between the musical rhythm STM task and the verbal order reconstruction STM task, the domain-generality of serial order mechanisms could be questioned. As no link was observed between the musical and verbal order reconstruction STM tasks, future studies will need to determine with more details whether the association between some serial order processing tasks in the two domains is due to the involvement of domain-general ordering mechanisms or whether theses associations only concern the rhythmic components shared between the tasks. This absence of association could have been due to suboptimal design of the musical serial order tone reconstruction task, as discussed later in the *Discussion*, but we cannot dismiss the possibility that the musical sequence information could be represented by additional, specific processes such as contour information (e.g., Gorin et al., 2016), especially when tones are presented in a monotonous rhythm which is untypical for musical information.

Our proposal that item information in musical STM is supported by the activation of domain-specific LTM representations is influenced by models of verbal STM that incorporated LTM activation-based principles, considering that the availability of linguistic representations in LTM is a critical determinant of STM capacities (e.g., Buchsbaum & D’Esposito, 2008; Cowan, 1995; Majerus, 2009, 2013; Oberauer & Hein, 2012). The influence of psycholinguistic knowledge on verbal STM performance has been demonstrated in many studies; all major psycholinguistic variables have been shown to influence STM such as semantic relatedness (Poirier & Saint-Aubin, 1995; Saint-Aubin & Poirier, 1999), lexicality (Hulme et al., 1991; Majerus & Van der Linden, 2003), lexical frequency or sublexical phonotactic probabilities (Gathercole et al., 1999; Hulme et al., 1997; Majerus, Van der Linden, Mulder, Meulemans, & Peters, 2004; Roodenrys, Hulme, Alban, Ellis, & Brown, 1994; Roodenrys et al., 2002; Thorn et al., 2005). In the musical field of STM, similar STM-LTM interactions have been reported, showing that musical STM capacities are dependent on the availability of timbral or tonal knowledge (Schulze et al., 2012; Siedenburg & McAdams, 2017).

### Is pitch short-term maintenance embodied in the vocal production system?

The presence of strong correlations between singing abilities assessed through immediate repetition of pitch/interval information and short-term recognition of tone lists for which no singing is required (see Table 3), opens the question of the codes underlying musical STM.^1^ Immediate vocal imitation of pitch and interval information is related to singing abilities. Singing can be viewed as a sensorimotor translation process requiring the mapping between a perceived target pitch and the phonatory motor codes needed to accurately produce the target pitch (Pfordresher, Halpern, & Greenspon, 2015). Thus, the presence of strong correlations between immediate vocal imitation and musical item STM tasks may indicate that the perceptual-motor processes involved in singing abilities is also involved during musical item recognition tasks. Since no correlations were observed between immediate vocal imitation tasks and pitch discrimination (immediate pitch imitation: BF_01_ = 3.72, *r* = –0.13; immediate interval imitation: BF_01_ = 3.31, *r* = –0.14), as well as interval discrimination tasks (immediate pitch imitation: BF_10_ = 1.02, *r* = –0.21; immediate interval imitation: BF_10_ = 1.31, *r* = –0.20), it is unlikely that the link between singing abilities and musical item recognition tasks is merely reflecting a musical perceptual component, in line with data showing that singing abilities are not necessarily linked to musical perception abilities (see, e.g., Pfordresher & Brown, 2007).

One explanation could be that in order to compare melodies, participants had to create an auditory image of the melodic target (Siedenburg & McAdams, 2017). This ability to form auditory images may also involve the motor codes underlying singing capacities (see Hutchins, Larrouy-Maestri, & Peretz, 2014; Hutchins, Zarate, Zatorre, & Peretz, 2010; Pfordresher & Mantell, 2014), which are necessary for ‘replaying’ the auditory image by subvocal reproduction of the auditory image (Greenspon, Pfordresher, & Halpern, 2017; Pfordresher & Halpern, 2013; Pfordresher et al., 2015). A recent study has shown that auditory-based rehearsal can be an efficient mechanisms to maintain tone sequences in STM (Nees, Corrini, Leong, & Harris, 2017), in line with the view that the perceptual-motor interface is a critical determinant of STM capacities (Hughes, Chamberland, Tremblay, & Jones, 2016). One possible interpretation of this pattern of results is that singing abilities, musical imagery, and the processes underlying rehearsal in musical STM tasks may be rooted in common sensorimotor mechanisms. More specifically, it could be argued that in order to perform a musical short-term recognition task, listeners translate the auditory input into an auditory-motor image that is maintained over time through subvocalization to allow performing the comparison judgement. Proficient singers would thus benefit from a more accurate sensorimotor translation of the auditory input, which in return could benefit to the comparison judgement. In that case, and according to evidence that poor-pitch singers exhibit less accurate imitation of pitches distant from their comfort pitch zone (Hutchins et al., 2014; Pfordresher & Brown, 2007), it is predicted that poor-pitch singers should have poorer musical STM capacity when the to-be-remembered material is composed of tones distant from their vocal comfort zone. However, further studies are needed to confirm this tentative interpretation and to determine more precisely the possible link existing between musical STM, musical auditory imagery, and sensorimotor coding processes.

### Limitations

Concerning the absence of associations between the verbal item STM composite score and the verbal immediate serial recall task, this absence of association is less surprising if we examine more closely the type of item STM processes that are recruited by the verbal immediate serial recall task. This task used word stimuli while the tasks entering the item STM composite score all used nonword stimuli. Indeed, nonword item STM tasks draw on sublexical phonological knowledge such as phonotactic knowledge about phoneme co-occurrences (Gathercole et al., 1999; Majerus, Martinez Perez, & Oberauer, 2012; Majerus et al., 2004) while word stimuli draw on lexical phonological and semantic knowledge (Hulme et al., 1991; see also Majerus, 2013). The dissociation between sublexical and lexical levels of item representations also is a classical finding in the psycholinguistic and neurolinguistic literature, showing that patients can be impaired at the lexical level (word repetition) while showing preserved sublexical processing (e.g., nonword repetition) and vice-versa, and this in both linguistic and verbal STM tasks (Majerus, 2018; Majerus, Norris, & Patterson, 2007). Hence, the absence of an association between the verbal item composite score and the item component of the immediate serial recall task may not be entirely surprising, as one of these scores reflects sublexical item processing/maintenance and the other reflects lexical item processing/maintenance. This is however a post-hoc interpretation that needs further exploration. Hence, the immediate serial recall task did not target the same item knowledge bases as the nonword item STM tasks that composed the verbal item composite score. The choice of using an immediate serial recall task for word lists had been made to allow for accurate assessment of both item and serial order STM performance in a single task. This would have been more difficult with nonword stimuli, given that serial recall for nonword lists leads to very low performance levels, as well as to phoneme exchange errors (Jefferies, Jones, Bateman, & Lambon Ralph, 2005) which are difficult to score in terms of item and order errors.

As regards the musical serial order reconstruction task, the overall correlation pattern indicates that this task may not have measured serial order STM capacities as purely and directe as initially intended. Although participants were requested to reconstruct the serial order of sequences composed of only three different tones in order to minimize musical item STM requirements, it appeared that the ability to discriminate basic musical units was a major determinant of performance on this task. Performances on that task were indeed moderately and strongly associated with pitch (BF_10_ = 3.47, *r* = 0.27) and interval (BF_10_ = 17.41, *r* = 0.32) discrimination abilities, respectively. In line with this view, Bayesian correlation analysis showed that the musical order reconstruction task strongly correlated with the tone list recognition task assessing retention of musical item identity (BF_10_ = 27.67, *r* = 0.34). At the same time, the tone list recognition task also correlated strongly with the verbal order reconstruction task (BF_10_ = 21.24, *r* = 0.33), probably reflecting the inherent serial aspect of the musical item recognition task. However, when predicting tone list recognition performance by the two discrimination and serial order reconstruction tasks (see Table 5), a separate analysis of the effect of each predictor revealed strong evidence that the verbal order reconstruction variable predicts scores from the tone list recognition task (BF_10_ = 22.90), while this was not the case for prediction of the scores of the musical order task (BF_01_ = 1.68). These results may be explained by the fact that the musical and verbal serial order reconstruction tasks contribute differently to the tone list recognition task assessing musical item retention, the musical order task being probably more strongly associated with tone identity processing and musical discrimination abilities than with serial order processing. If this is true, this may explain the fact that the musical order STM task was not associated with the rhythm STM task. Since musical discrimination abilities appear to have driven performance in the musical order reconstruction tasks, it is likely that this task had a strong requirement on musical discrimination capacities despite the fact that initially this task had been designed to mainly assess serial ordering abilities and that we used distant tones (as in Williamson et al., 2010) to avoid confusion between proximal tones.

**Table 5:** Results of the top-down Bayesian regression analysis for tone lists recognition task.

Predictor of interest	BF_10_	BF_01_

Pitch discrimination	11.04	0.09
Interval discrimination	0.57	1.74
Serial order reconstruction for digits	22.90	0.04
Serial order reconstruction for tones	0.59	1.68

Legend: BF_10_ relates the evidence for the full model relative to the same model without the predictor of interest (i.e. the evidence in favor of the predictor of interest) while BF_01_ represents the evidence for the model without the predictor of interest relative to the full model (i.e. the evidence against the predictor of interest).

## Conclusion

To conclude, the present exploratory study provides preliminary results suggesting that, at some level, similar cognitive principles could support the maintenance of verbal and musical stimuli in STM. This seems particularly the case for sequential processing (see also Gorin et al., 2016, 2018a, 2018b) while maintenance of memoranda identity appears to rely on domain-specific representations (see Albouy et al., 2018; Tillmann et al., 2016). One possibility to accommodate evidence for both domain-specific and domain-general processes in verbal and musical STM would be to consider a general architecture where STM maintenance results from the interplay between modality-specific representations—required to process item information in verbal and musical domains of STM—and amodal serial, temporal order processes involved in the processing of sequential information in the two STM domains. More generally, our results suggest that the item versus order structure of verbal STM could be extended to the musical domain, by integrating this distinction into a broader STM structure characterized by domain-specific processes for the representation of item information, and domain-general processes for and the representation of order information. However, in order to support more strongly this view, further studies are needed to determine with more details the nature of item and order representations in the musical domain, as well as the tasks the best suited to study these two components. Future studies will also need to determine whether amodal, temporal codes are the only codes, or whether domain-general temporal codes may co-exist with additional, domain-specific serial order representational processes.

## Data Accessibility Statement

The statement would say “All relevant data are available through the Open Science Framework (https://osf.io/hwrms/).”

## Footnotes

We also observed a link between tone list immediate serial recognition (global score) and immediate imitation of pitch-interval (BF_10_ = 311.94, *r* = –0.40, representing decisive evidence) and single-pitch (BF_10_ = 9.15, *r* = –0.30, representing nearly strong evidence).
